# Supplementary material for: Genetic Inactivation of Free Fatty Acid Receptor 3 Impedes Behavioral Deficits and Pathological Hallmarks in the APPswe Alzheimer’s Disease Mouse Model
Source: Int J Mol Sci. 2022 Mar 24;23(7):3533. doi: 10.3390/ijms23073533 (PMC8999053; doi:10.3390/ijms23073533)
Supplement: Supplementary file 1 [file ijms-23-03533-s001.zip › ijms-1642988-supplementary.pdf]

Detailed Supplemental Methods and Materials

Animal genotyping

PCR genotyping used the primer set 5'-CAC ACT GCT CGA TCC GGA ACC CTT and 5'-GAG AAC TGT CTG GAA AAC GCT CAC to identify the mutant Ffar3 allele, 5'-CGA CGC CCA GTG GCT GTG GAC TTA and 5'-GTA CCA CAG TGG ATA GGC CAC GC to detect the WT allele [84]. To identify the mutant APP allele we used 5´GTT GAG CCT GTT GAT GCC CG and 5´GTT GAG CCT GTT GAT GCC CG, and to detect the WT allele 5´AAG CGG CCA AAG CCT GGA GGG TG with 5´GTG GAT AAC CCC TCC CCC AGC CTA GAC CA.

Fear conditioning-based memory induction protocol

To induce the immediately early genes, all the animals were placed under a cognitive stimulus based on a fear conditioning test as described previously [86]. The conditioning box is housed inside a soundproof chamber, which minimized external stimulation during the conditioning and retention tests. The box was provided with a house light that supplied dim illumination and a floor grid through which foot shocks could be administered. The mice were placed in the conditioning chamber for 2 min before the onset of a tone at 2800 Hz, 85 dB (conditioned stimulus, CS), which lasted for 30 s. The last 2 s of the CS was paired with a 0.3 mA foot shock (unconditioned stimulus, US). After the shock and 10 s of resting the same CS-US was delivered three consecutive times. Finally, 30 s after the last pair of CS-US, the mice were returned to their home cages. The conditioning apparatus was controlled by the experimenter with specific software (Packwin, Panlab S.L., Spain) running on a PC. Two hours after the fear conditioning test, the animals were killed and the brains removed for biochemical studies.

Immunohistochemistry

Nine free floating tissue sections comprising the hippocampal formation of three animals per group were processed for immunohistochemistry. Brain sections were washed (3 × 10 min) with a solution buffer containing PB 0.125 M (pH 7.4), 0.5% Triton X-100 and 0.1% Bovine serum albumin (BSA) (Sigma-Aldrich, Saint Louis, Mo, USA). After washing, sections were treated with methanol and H_2O_2 to inhibit endogenous peroxidase activity and incubated in 70% formic acid for 7 min to expose the epitope. Sections were incubated overnight with primary antibody 6E10 (anti-Aβ amino acids 1-17) (1:200, Millipore Corporation, Billerica, MA, USA) at 4°C. After washing, sections were incubated sequentially with biotinylated goat anti-mouse secondary antibody (1:500 DakoCytomation, Glostrup, Denmark) for 2 h, an ABC kit

immunoassay detection systems (Vector, Burlingame, CA, USA) for 90 min, and developed with 3,3'-diaminobenzidine (DAB) solution (Peroxidase substrate kit, Vector, Burlingame, CA, USA). Sections were then washed in distilled water before dehydrating and mounting in DPX (*BDH* Gurr, Poole, UK).

Immunoblotting

Protein samples were mixed with 6X Laemmli sample buffer [47% (v/v) glycerol, 0.6M Dithiothreitol, 12% (w/v) SDS, 0.08M Tris and bromophenol blue] resolved onto SDS-polyacrylamide gels [93] and transferred to nitrocellulose membrane. The membranes were blocked with 5% milk, 0.05% Tween-20 in phosphate buffered saline (PBS) or tris-buffered saline (TBS) followed by overnight incubation with the following primary antibodies: rabbit polyclonal anti-Arc (1:1000, Millipore Corporation, Billerica, MA, USA), rabbit polyclonal anti-c-Fos (1:200, Santa Cruz Biotechnology, CA, USA), rabbit polyclonal anti-pCREB (1:500, Upstate-Millipore, Temecula, CA, USA), rabbit polyclonal anti-CREB (1:1000, Cell Signaling Technology, Beverly, MA, USA), rabbit polyclonal anti-pGluR1 (1:1000, Millipore Corporation, Billerica, MA, USA), rabbit polyclonal anti-GluR2/3 (1:1000, Millipore Corporation, Billerica, MA, USA), rabbit polyclonal anti-BDNF (1:500, Santa Cruz Biotechnology, CA, USA), rabbit polyclonal anti-pGSK3-Ser^{9} (1:1000, Cell Signaling Technology, Beverly, MA, USA), mouse monoclonal anti-pGSK3β-Tyr^{216} (1:1000, BD Transduction Laboratories, Lexington, KT, USA), rabbit polyclonal anti-GSK3 (1:1000, Santa Cruz Biotechnology, Santa Cruz, CA, USA), rabbit polyclonal anti-p35/p25 (1:1000, Cell Signaling Technology, Beverly, MA, USA), mouse monoclonal anti-ptau AT8 (1:1000, Thermo Fisher Scientific, Rockford, USA), mouse monoclonal anti-tau (1:5000, clone Tau46, Sigma-Aldrich, St. Luis, MO, USA), rabbit polyclonal anti-MAP-2 (1:1000, Millipore Corporation, Billerica, MA, USA), mouse monoclonal anti-PSD95 (1:1000, Millipore Corporation, Billerica, MA, USA), mouse monoclonal anti-pCaMK II (1:500, Millipore Corporation, Billerica, MA, USA), mouse monoclonal anti-CaMK II (1:1000, Millipore Corporation, Billerica, MA, USA), mouse monoclonal anti-IDE (1:1000, Abcam, Cambridge, UK), and mouse monoclonal anti-β actin (1:2000, Sigma-Aldrich, St. Louis, MO, USA) in the corresponding buffer. Following two washes in PBS/Tween-20 or TBS/Tween-20 and one PBS or TBS alone, immunolabelled protein bands were detected by using HRP-conjugated anti-rabbit or anti-mouse antibody (1:5000, Santa Cruz Biotechnology, Santa Cruz, CA, USA) following an enhanced chemiluminescence system (ECL, GE Healthcare Bioscience, Buckinghamshire, UK), and autoradiographic exposure to Hyperfilm ECL (GE Healthcare Bioscience). Quantity One software v.4.6.3 (Bio-Rad, Hercules, CA, USA) was used for quantification.

For the analysis of APP-derived CTFs, aliquots of the protein extracts were mixed with XT sample buffer plus XT reducing agent or Tricine sample buffer (Bio-Rad, Hercules, CA, USA) and boiled for 5 min. Proteins were separated in a Criterion™ precast Bis-Tris 4–12% gradient precast gel (Bio-Rad, Hercules, CA, USA) and transferred to nitrocellulose membranes. The membranes were blocked with 5% milk, 0.05% Tween-20 in TBS followed by overnight incubation with rabbit polyclonal anti-APP C-terminal (amino acids 676–695) (1:2000, Sigma-Aldrich, St. Louis, MO, USA).

RNA extraction and Real Time PCR

Total RNA was extracted from the brain hippocampus by Chomczynski and Sachi's method [94] with the TRI reagent (Sigma-Aldrich, MO, USA). Briefly, cells were washed with PBS and lysed with 1 mL TRI reagent. After 5 min of incubation at room temperature, the lysate was mixed vigorously with 0.2 mL of chloroform. The sample was then centrifuged at 12.000x*g* for 15 min at 4°C, and the supernatant was recovered and placed in a fresh tube containing 0.5 mL isopropanol. After incubating for 10 min at room temperature, the RNA pellet was obtained by centrifugation at 12.000x*g* for 10 min at 4°C. The pellet was washed in 1 mL ethanol (75%) and, once all the ethanol had been removed, it was dissolved in 30 µL diethylpyrocarbonate-treated water (DEPC) (Sigma-Aldrich, MO, USA).

2 µg of total RNA obtained was used as a template to synthesize cDNAs with the SuperScript® III First-Strand Synthesis System for real-time PCR (RT-PCR) (Invitrogen, Life technologies, Carlsbad, CA, USA). RT-PCR assays were then performed in triplicate on these cDNAs in the presence of the PCR Master Mix (Power SYBR®Green, Applied Biosystems, Warrington, UK) to detect the amplification products. Samples were analyzed simultaneously for ribosomal protein 36B4 mRNA as an internal control using an ABI Prism 7300 sequence detector (Applied Biosystems, Foster City, CA, USA). The data were analyzed using Sequence Detection software v. 3.0. (Applied Biosystems). The primer sequences for quantitative PCR are indicated in Table 1.

Table S1. Primer sequences used for RT-PCR.

Ffar1 forward	GCTGCTGCTCTGCGTAGGA
Ffar1 reverse	CAGCGGATTAAGCACCACACT
Ffar3 forward	ACCAGTGATTGCCGGAGAAG
Ffar3 reverse	CCACCATGGATACAGGCCC

Ffar2 forward	GGCTTTCCCCGTGCAGTAC
Ffar2 reverse	CCAGAGCTGCAATCACTCCAT
Neprilysin forward	GCAGCCTCAGCCGAAACTAC
Neprilysin reverse	CACCGTCTCCATGTTGCAGT
36B4 forward	AACATCTCCCCCTTCTCCTT
36B4 reverse	GAAGGCCTTGACCTTTTCAG
